# Medical Convention in Illinois and Wisconsin

**Published:** 1846-06-01

**Authors:** 


*Medical Convention in Illinois and Wisconsin.*—A Convention of the medical profession in the Northern portion of Illinois and the Southern portion of Wisconsin has recently been held, preparatory to a permanent organization. Another meeting was appointed for the 3d Tuesday in May, to adopt by-laws, elect officers, &c. It is to be purely a voluntary association, and it is distinctly declared by the Convention, that no legislative protection is to be *asked for.* The qualifications for membership are to be a degree of doctor in medicine, a diploma, or license from some properly constituted association, or a satisfactory examination by a board of censors to be elected by the society.

Hitherto there has existed no medical organization either in Illinois or Wisconsin, nor are there any laws in those sections of our country regulating the practice of medicine and surgery, *except for the recovery of damages in cases of mal-practice.* We are glad to see this action among our brethren of the North West, and have no doubt it will be productive of great and lasting benefits.

